# Osmotic Stress Adaptation of Poultry-Associated *Salmonella* Infantis and Its Implications for Food Safety

**DOI:** 10.3390/foods15111938

**Published:** 2026-05-31

**Authors:** Gabriel I. Krüger, Ana Oviedo, Coral Pardo-Esté, Nicolás Avilés-Núñes, Sofía Quintana, Alejandro A. Hidalgo, Javiera Álvarez, Francisca Urbina, Catalina Kusch, Katterinne N. Mendez, Jorge Olivares-Pacheco, Luis Alvarez-Thon, Francisco Remonsellez, Juan Castro-Severyn, Claudia P. Saavedra

**Affiliations:** 1 Facultad de Ingeniería y Negocios, Universidad de las Américas, Sede Providencia, Manuel Montt 948, Santiago 7500975, Chile; gkruger@udla.cl; 2 Laboratorio de Microbiología Molecular, Facultad de Ciencias de la Vida, Universidad Andres Bello, Republica 330, Santiago 8370186, Chile; acob11doctorado@gmail.com (A.O.); cpardoeste@gmail.com (C.P.-E.); nicolas.aviles0170@gmail.com (N.A.-N.); qsofiae@gmail.com (S.Q.); jalvarezm1011@gmail.com (J.Á.); fran.urbina17@gmail.com (F.U.); ckusch03@gmail.com (C.K.); mendez.katterinne@gmail.com (K.N.M.); jsevereyn@gmail.com (J.C.-S.); 3 Escuela de Química y Farmacia, Facultad de Medicina, Universidad Andres Bello Sazie 2320, Santiago 8370149, Chile; alejandro.hidalgo@unab.cl; 4 Allergic Inflammation Group, Department of Immunology and Microbiology, University of Copenhagen, DK-1165 Copenhagen, Denmark; 5 Grupo de Resistencia Antimicrobiana en Bacterias Patógenas y Ambientales, (GRABPA), Instituto de Biología, Pontificia Universidad Católica de Valparaíso, Valparaiso 2373223, Chile; jorge.olivares@pucv.cl; 6 Centro de Investigación en Ingeniería de Materiales (CIIMAT), Facultad de Ingeniería y Arquitectura, Universidad Central, Santa Isabel 1186, Santiago 8330601, Chile; luis.alvarez@ucentral.cl; 7 Laboratorio de Microbiología Aplicada y Extremófilos, Departamento de Ingeniería Química, Universidad Católica del Norte, Angamos 0610, Antofagasta 1270709, Chile; fremonse@ucn.cl

**Keywords:** *Salmonella* Infantis, osmotic stress, biofilm, poultry industry, transcriptomics

## Abstract

*Salmonella enterica* serovar Infantis, an important zoonotic pathogen with increasing prevalence in the poultry industry, often persists despite rigorous disinfection. This study characterized the transcriptomic response of the multidrug-resistant *Salmonella* Infantis strain SE016, isolated from a poultry plant, to osmotic stress, a condition frequently induced by the use of industrial disinfectants. Phenotypic assays demonstrated that stress induced by 15% sucrose simulated osmotic stress, producing a drastic reduction in flagellar motility and a significant increase in biofilm formation in SE016, compared with a susceptible control strain. RNA-seq analysis indicated that SE016 undergoes coordinated transcriptional changes consistent with altered metabolic activity under osmotic stress. Key mechanisms include metabolic braking through repression of tricarboxylic acid (TCA) cycle genes (*icd*, *mdh*) and induction of anaerobic nitrate respiration (*narGHI*, *narZWV*) as an energy contingency. Furthermore, SE016 showed increased expression of genes involved in osmoprotectant uptake, including the *proU* transport system and endogenous trehalose synthesis (*ostAB*) while repressing proline degradation (*putA*). Furthermore, robust biofilm formation was observed despite repression of the master regulator *csgD*. This was mediated by the CsgD-independent induction of the diguanylate cyclase *adrA*, activating cellulose synthesis (*bcs*). These results suggest that pathways associated with the OmpR/EnvZ two-component system may contribute to energy balance and persistence-related phenotypes under industrial-like stress conditions.

## 1. Introduction

*Salmonella enterica* serovar Infantis (*S.* Infantis) is increasingly recognized as a major zoonotic pathogen, with a global rise in its prevalence in human salmonellosis cases in recent decades [1]. In fact, it has been identified as the second most prevalent zoonotic pathogen in the European Union, with a reported 16.9% increase in confirmed human cases in 2023 compared to 2022 [2]. This growing incidence not only highlights the magnitude of the associated public health problem but may also be associated with increased environmental persistence, potentially linked to stress-tolerance traits and/or limitations in current cleaning and disinfection practices, specifically those involving the use of sodium hypochlorite, quaternary ammonium compounds, peracetic acid, and glutaraldehyde. They might even be linked to inadequate current standard cleaning and control measures.

The poultry industry, particularly the meat production line, is considered a primary source of *S.* Infantis contamination in various regions, including the United States, Europe, and Latin America [3,4]. *S.* Infantis is frequently isolated from chicken meat sampled from production lines, indicating recurrent and widespread contamination in farms. One of the most striking features of *S.* Infantis isolates is the increasingly common presence of emerging mega-plasmids (pESI or pESI-like), which carry genes for adaptation, virulence, and antimicrobial resistance [5].

The pESI plasmid confers a series of advantageous traits that contribute to the adaptation and survival of *S.* Infantis against various stress factors, including multidrug resistance; disinfectant resistance; increased tolerance to heavy metals; augmented virulence; enhanced biofilm formation capacity; and greater tolerance to thermal, acid, oxidative, and osmotic stress [6,7].

The dissemination of pESI-bearing strains appears to be driven by poultry industry practices, in which the intensive use of antimicrobials and disinfectants may contribute to selection pressures favoring resistant *S*. Infantis variants [8]. Previous studies have reported that conjugative transfer of pESI-like plasmids may be influenced by environmental factors, including temperature, oxidative stress, osmolarity, and biofilm formation. These observations raise the possibility that stress conditions in poultry environments could favor both persistence of pESI-bearing strains and horizontal gene transfer [7]. The relevance of these systems transcends mere transcriptomic observation, as they are grounded in regulatory hierarchies whose mechanistic causality has been experimentally validated.

The environmental recurrence of *S.* Infantis in poultry farms, even after rigorous cleaning and disinfection protocols, is largely attributed to its ability to form biofilms [9,10]. This survival strategy is fundamental to contamination of key surfaces such as water systems, feed lines, and processing equipment within the production line. Biofilm formation in *Salmonella* depends on key molecular determinants, such as curli fimbriae and cellulose, encoded by the *csg* and *bcs* operons, respectively [8,11,12]. Furthermore, the presence of the pESI plasmid has been directly correlated with an increased capacity for biofilm formation, creating synergy in which the genetic resistance conferred by the plasmid is combined with the physical protection of the biofilm [10].

Disinfection procedures in the poultry industry expose bacteria to multiple stresses, including chemical injury, oxidative stress, and, in some settings, desiccation or elevated osmolarity associated with residues or drying surfaces. Sublethal exposures may therefore create heterogeneous microenvironments that influence bacterial survival. The connection between this stress and the survival capacity of *S.* Infantis is critical, given that pESI-plasmid-carrying strains exhibit greater tolerance to osmotic stress [6]. Various disinfectants used in poultry processing generate osmotic stress through solute imbalances, leading to cellular dehydration [13,14]. Agents such as concentrated chloride solutions or strong acids act directly by creating hyperosmotic environments that damage proteins, DNA, and membrane fluidity [15,16]. Others, including sodium hypochlorite and quaternary ammonium compounds, induce this stress secondarily by compromising cellular permeability [17]. In pathogens such as *Salmonella enterica*, these simultaneous pressures trigger adaptive responses that enhance survival and promote biofilm formation [18,19].

Under osmotic stress and desiccation, *Salmonella* deploys a repertoire of adaptive strategies shaped by selective pressures that require metabolic reconfiguration; alterations in membrane fluidity; and the accumulation of compatible osmolytes such as trehalose, proline, and betaine [20]. These selective pressures trigger physiological responses that frequently confer cross-protection, increasing the pathogen’s resistance to thermal barriers, radiation, and biocidal agents [21]. In industrial processing, sublethal exposure to solute imbalances can pre-adapt bacterial communities, enhancing their ability to establish infections by temporarily suppressing growth and modifying virulence factors [22].

*Salmonella*, in general, responds to changes in osmolarity by accumulating compatible solutes and modulating gene expression through key regulators like the sigma factor RpoS and the two-component system EnvZ-OmpR [23]. The OmpR regulator provides a direct molecular link between osmotic sensing and biofilm regulation, as it controls the expression of *csgD*, the master regulator of curli and cellulose production [24]. However, this presents an apparent contradiction under high-osmolarity conditions, as OmpR has been shown to repress *csgD* expression in these conditions [15,25,26]. This suggests the existence of alternative or complementary regulatory mechanisms that allow *S.* Infantis to overcome this repression and build a protective biofilm.

In this context, the bacterial second messenger cyclic di-GMP (c-di-GMP) emerges as a prime candidate for orchestrating this complex adaptive response [27,28]. c-di-GMP is a master regulator that controls bacterial lifestyle transitions; increased levels promote biofilm formation by activating cellulose and curli synthesis and the sessile state, while decreased levels favor motility [28,29]. The intracellular concentration of c-di-GMP is finely regulated by a set of enzymes, diguanylate cyclases (DGCs) and phosphodiesterases (PDEs), which respond to various environmental and intrinsic signals [30]. Beyond being a simple biofilm switch, c-di-GMP can redirect carbon metabolism to favor extracellular polymeric substance (EPS) production and modulate the expression of a wide range of genes [31,32].

The relevance of these systems transcends mere transcriptomic observation, as they are grounded in regulatory hierarchies whose mechanistic causality has been experimentally validated. The EnvZ/OmpR system acts as the primary sensor of cytoplasmic signals such as pH and osmolality, directly linking physical stress to a massive reprogramming of the bacterial transcriptome and the control of central metabolism. Specifically, OmpR has been shown to directly regulate key promoters, such as *gltA*, to maintain energy balance during internal acidification [23]. Concurrently, the transition to a sessile lifestyle is centrally governed by the AdrA-c-di-GMP axis, a validated pathway that induces cellulose biosynthesis and extracellular matrix maturation in *Salmonella* [33]. These precedents establish that variations in the expression of these genes under stress are not isolated correlational events, but the execution of a structured genetic program for persistence.

Although the individual roles of key components and genetic determinants that enable persistence and biofilm formation in *S*. Infantis are established, transcriptomic data on osmotic stress responses in poultry-derived *S.* Infantis isolates are lacking. Moreover, the relationship between osmotic adaptation and biofilm persistence remains unknown, as does the precise role of the c-di-GMP signaling network in overcoming regulatory contradictions. Therefore, the objective of this study was to evaluate the biofilm-forming capacity of a poultry-derived *S*. Infantis isolate, characterize its early transcriptional regulation in response to biofilm-inducing conditions via RNA-seq, and investigate whether c-di-GMP-associated pathways contribute to the osmotic stress response of the poultry-derived *S*. Infantis isolate. Addressing these questions is fundamental for developing future control strategies aimed at dismantling the mechanisms that make *S.* Infantis such a successful pathogen.

## 2. Materials and Methods

### 2.1. Bacterial Strain and Growth Conditions

In this study, the *Salmonella* Infantis isolate SE016 (Bioproject: PRJNA890630; SAMN42896070) was used, which was obtained and characterized in previous studies [18,34,35]. In those studies, strain SE016 was categorized as a resistant strain from the poultry meat production line and was isolated from a poultry farm environment. As a comparative control, we used *Salmonella* Infantis strain SARB27 (ATCC BAA-1675), a rare multilocus enzyme electrophoresis (MLEE) variant (In3) isolated in Senegal prior to 1993 [36]. SARB27 was selected for its significant genotypic divergence from contemporary industrial lineages, specifically for its lack of the pESI-like megaplasmid and other mobile genetic elements associated with modern multidrug resistance [35]. Functionally, this strain has been characterized as highly sensitive to osmotic stress and industrial disinfectants [18].

Osmotic stress conditions were induced using Glucose Minimum Medium (MgM) as described by Chakraborty & Kenney (2018) [23]. Briefly, the medium was buffered with 100 mM Tris at pH 7.2 and supplemented, when indicated, with 15% (*w*/*v*) sucrose. The medium contained 7.5 mM (NH_4_)_2_SO_4_, 5 mM KCl, 0.5 mM K_2_SO_4_, 1 mM KH_2_PO_4_, 10 mM MgCl_2_, 2 mM glucose, and 0.1% casamino acids. Each strain was grown in LB medium for 16 h at 37 °C with agitation at 150 rpm. An aliquot of the culture (1:100) was used to inoculate MgM (pH 7.2), and the mixture was incubated for 24 h at 37 °C with agitation at 150 rpm. Subsequently, the cultures were subcultured (1:50) into osmotic medium (pH 7.2 + 15% sucrose) or control medium (pH 7.2) for the duration of the assays. The use of 15% (*w*/*v*) sucrose provides a defined and reproducible hyperosmotic condition that reduces water activity and challenges bacterial osmotic homeostasis. This approach allows controlled activation of osmotic stress regulatory systems (e.g., EnvZ/OmpR) without introducing additional chemical toxicity associated with disinfectants. All physiological assays were performed with 3 biological replicates, and measurements were taken in 3 technical replicates per biological replicate.

### 2.2. Biofilm Growth Kinetics

To observe the biofilm formation of both *S.* Infantis strain communities, SE016 and SARB27, we measured their biofilm growth kinetics. Biofilm formation was determined by optical observation following crystal violet (CV) staining in 24-well microdilution plates (SPL, catalog 30024), as described by Krüger & Urbina et al. (2025) [18]. Briefly, a 1:10 dilution of the bacterial culture was prepared in MgM medium with and without 15% sucrose treatment. The plates were incubated without agitation at 37 °C for 4, 8, 12, 16, 20, and 24 h. After the incubation period, the medium was removed using a vacuum pump, and the biofilm was subsequently washed three times with sterile 1X PBS. Fixation was performed in an inverted position at 60 °C for 1 h. Staining was conducted with 0.1% CV and incubated for 15 min at room temperature. Excess CV was removed using a vacuum pump and washed once with sterile distilled water. Finally, the biofilm was solubilized in 33% acetic acid for 15 min at room temperature without movement. Absorbance was quantified at 570 nm using a Tecan Infinite 200 PRO reader (Tecan Life Sciences). For each isolate, biofilm formation and its intensity were assayed in three biological replicates, with five technical replicates per plate (*n* = 15). Biofilm formation was interpreted as follows: the cutoff optical density (ODc) was defined as the mean absorbance of the negative control wells, plus their standard deviation, three times. Isolates were then classified based on their mean absorbance (OD_570 nm_):

0. Non-forming isolates: OD_570 nm_ ≤ ODc.

1. Weak biofilm formation isolate: ODc < OD_570 nm_ ≤ 2xODc.

2. Moderate biofilm formation isolate: 2xODc < OD_570 nm_ ≤ 3xODc.

3. Strong biofilm formation isolate: 3xODc < OD_570 nm_ ≤ 4xODc.

4. Very strong biofilm formation isolate: OD_570 nm_ > 4xODc.

### 2.3. Motility Assays

Each strain was subjected to a motility test using the 0.3% agar motility plate method (3 g/L of agar; 10 g/L of tryptone; 5 g/L of NaCl) with and without 15% sucrose. A volume of 2 μL was inoculated into the center of the plates (90 mm) from each culture incubated for 4, 8, and 12 h. The plates were then incubated at 37 °C for 18 h. The area (cm^2^) of the motility zone was measured using ImageJ software (v1.80_345).

### 2.4. Survival Challenge Against Exposure to Industrial Disinfectants

S. Infantis strains were cultured in Luria–Bertani (LB) broth for 18 h at 37 °C with shaking at 150 rpm. Subsequently, the cultures were diluted 1:100 in MgM medium and incubated for 24 h at 37 °C and 150 rpm. Bacterial density was estimated by measuring OD_600_. For the cultures, the average OD_600_ was approximately 0.3 (6 × 10^8^ CFU/mL). For biofilm formation, the bacterial cultures were diluted 1:10 in MgM medium in 24-well plates, reaching a final volume of 1 mL per well. The plates were incubated at 37 °C for 24 h without agitation. Then, the planktonic fraction was removed, and the adherent biofilms were washed twice with sterile 1X PBS. Subsequently, the previously prepared disinfectant solutions were added at the highest concentration and maximum exposure time specified by the manufacturer (Table 1). Once the exposure time was complete, the disinfectants were removed, and the biofilms were washed twice with sterile 1X PBS. To recover the cells associated with the biofilm, 1 mL of sterile 1X PBS was added per well, and two 30 s pulses of low-frequency sonication were applied (Elma, Singen, Germany). Bacterial viability was determined by CFU counting. Recovered suspensions were serially diluted, and 4.5 μL aliquots were spot-plated onto LB agar plates, which were incubated at 37 °C for 12 h. The experiment was performed in biological triplicate and technical duplicate (n = 24 per strain and condition).

### 2.5. Determination of Minimum Inhibitory Concentration of Industrial Disinfectants

The minimum inhibitory concentration (MIC) was determined in MgM medium. Each isolate was cultured in 30 mL of LB medium for 18 h with agitation at 150 rpm and 37 °C. After incubation, the isolates were subcultured (1:100) in 30 mL of MgM medium for 24 h until an OD600 nm of between 0.3 and 0.4 was reached (pre-inoculum) Then, an aliquot was taken to dilute the culture in MgM to approximately 0.03 (inoculum). In parallel, dilutions of the disinfectants (10% Quaternary Ammonium—10% Sodium Hypochlorite—15% Peracetic Acid—2% Glutaraldehyde) were prepared from a stock solution of 4.5 mL/L–2 mL/L–2 mL/L–3 mL/L–10 mL/L, respectively. Six serial dilutions were performed at a dilution factor of two. The MIC was determined in sterile 96-well plates (Nunc™ Microwell 96-Well Microplate, 167013, Thermo Scientific, Waltham, MA, USA), where 190 µL of the inoculum and 10 µL of each disinfectant dilution were added. This was performed for each strain with each disinfectant dilution in triplicate biological and duplicate technical samples (n = 24); additionally, the condition without disinfectant was observed as a positive growth control. In all conditions three consecutive washes were performed with sterile PBS to eliminate remnants of the compounds. After setting up the 96-well plate, it was incubated for 20 h without agitation at 37 °C. Subsequently, absorbance was measured at OD600 nm using an Infinite 200 PRO plate reader (Tecan Life Sciences, Männedor, Switzerland). The MIC was defined as the concentration corresponding to the turbidity-free well adjacent to the one showing growth.

### 2.6. Determination of c-di-GMP Concentration During Biofilm Formation

Quantification of c-di-GMP levels in the strains was performed by taking an aliquot of the planktonic state cultures at 8, 16, and 24 h of incubation. This was conducted using the Cyclic-di-GMP Assay kit (Catalog No. 200-100, Lot #200724, Lucerna Technologies, Brooklyn, NY, USA) according to the manufacturer’s instructions. The fluorescence signal was measured in a plate reader Synergy H1 Microplate Reader (BioTek, Winooski, VT, USA), using excitation/emission wavelengths of 469/501 nm.

### 2.7. RNA Extraction/Isolation and Sequencing

Strains SE016 and SARB27 were cultured in MgM pH 7.2 for 24 h, then subcultured (1:50) into MgM pH 7.2 and MgM pH 7.2 supplemented with 15% (*w*/*v*) sucrose for 5–6 h until reaching an optical density at 600 nm (OD_600_) of approximately 0.4. RNA was extracted and isolated using the E.Z.N.A. Total RNA Kit I (Omega Bio-Tek, R6834, Norcross, GA, USA), as specified by the manufacturer. Total RNA was sequenced at SeqCenter (Pittsburgh, PA, USA). Ribosomal RNA (rRNA) depletion sequencing was performed on the NovaSeq 6000 Illumina platform, generating independent 150 bp paired-end libraries. Each treatment for each strain was performed in triplicate, resulting in three control libraries and three libraries treated with 15% (*w*/*v*) sucrose for both the SE016 and SARB27 strains. For each condition, 3 biological replicates were used per strain.

### 2.8. RNA-Seq Analysis

On average, 16.9 million reads were obtained, representing a 530× coverage. Raw data quality control was performed using FastQC v0.12.1, followed by filtering and trimming with Trim-Galore v2.0 (parameters: minimum length 50 bp, 0 N’s, and <Q25 threshold). Gene expression levels were estimated by mapping reads against their respective reference genomes: SE016 reads were mapped against the *S. enterica* Infantis SE016 genome (Bioproject: PRJNA890630; SAMN42896070), and SARB27 reads against the *S. enterica* Infantis SARB27 genome (GenBank: CM001274.1), using STAR v2.7.10b [37]. The count matrix results were used to estimate differential gene expression by implementing the DESeq2 Bioconductor R package [28]. Expression values are represented as log_2_ after filtering for |LFC| ≥ 1 and adjusted *p*-value ≤ 0.01. RNA-seq data is available in the NCBI SRA database under accession number PRJNA1422429. GO/KEGG enrichment analysis was carried out using the clusterProfiler v4.20, topGO v2.64.0, Bioconductor (v3.23) R packages.

## 3. Results

### 3.1. Differential Susceptibility of SE016 and SARB27 to Industrial Disinfectants in Sessile and Planktonic States

Survival challenges against chemical agents revealed a significant phenotypic divergence between the analyzed strains. In the sessile state, the SE016 strain exhibited a robust growth capacity in the presence of oxidizing disinfectants, compared with the reference strain SARB27 (Supplementary Figure S1). Specifically, in sodium hypochlorite assays, SARB27 showed no detectable growth, whereas SE016 proliferated in a fraction of the tested conditions (*p* < 0.01). The most critical disparity was observed with peracetic acid, where SE016 exhibited frequent growth events, whereas SARB27 showed almost total inhibition (*p* < 0.0001). In contrast, non-oxidizing agents such as quaternary ammonium and glutaraldehyde severely inhibited both strains, with no clear differences in resilience under these conditions. These biofilm findings contrast with the Minimum Inhibitory Concentration (MIC) profiles obtained in the planktonic state (Supplementary Table S1). In suspension, SE016 showed no competitive advantage; in fact, it exhibited greater susceptibility to sodium hypochlorite (MIC of 25 mg/L) than SARB27 (50 mg/L). For compounds such as peracetic acid and Sodium hypochlorite, both strains exhibited comparable MIC ranges (75–37.75 mg/L and 75–18.75 mg/L, respectively). In the case of glutaraldehyde, SE016 even showed slightly higher sensitivity (6.25–12.5 mg/L) than the reference (12.5–25 mg/L).

### 3.2. Effect of Osmotic Stress on Motility and Biofilm Formation in SARB27 and SE016 Strains

**Figure 1:**
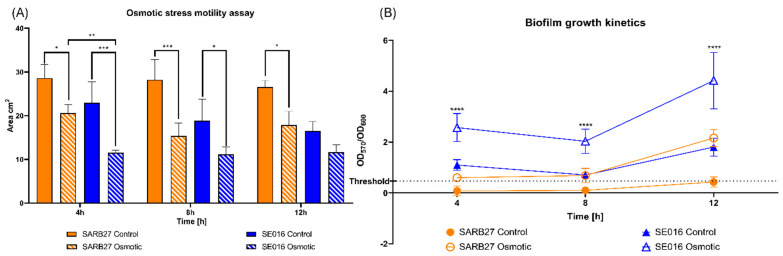
The effect of osmotic stress on motility and biofilm formation in SARB27 and SE016 strains. (**A**) Motility assay on semi-solid agar plates. Bacterial migration area (cm^2^) was measured at 4, 8, and 12 h post-inoculation for SARB27 and SE016 strains under control and osmotic stress conditions. Biofilm formation kinetics. (**B**) Attached biofilm biomass was quantified by crystal violet staining, normalized to total cell density at the same time points. Dotted line: positive biofilm formation threshold. Statistical analysis was performed using two-way ANOVA with Tukey’s correction for multiple comparisons. Asterisks: significant differences (* *p* < 0.05, ** *p* < 0.01, *** *p* < 0.001, **** *p* < 0.0001).

To evaluate the impact of osmotic stress on *Salmonella* Infantis strains, motility assays and biofilm formation kinetics were performed. The bacterial migration area in semi-solid agar was quantified at 4, 8, and 12 h post-inoculation for strains SARB27 and SE016, under both osmotic stress and control conditions (Figure 1A). The observation of differential motility between strains and conditions suggests specific adaptive mechanisms. Osmotic stress significantly reduced motility compared with control conditions. When considered together with the increased biofilm biomass observed under the same conditions, these findings support an inverse relationship between motility and surface-associated growth.

Biofilm formation kinetics were determined by crystal violet staining (absorbance at 570 nm) and normalized to total cell density (absorbance at 600 nm) at the same time points (4, 8, and 12 h). Figure 1B illustrates the attached biofilm biomass. A threshold for considering positive biofilm formation was established, indicated by the dotted line. The ability of *Salmonella* to form biofilms under osmotic stress is a critical factor in its persistence in environments such as poultry production lines, where such stress is common. The observed variability between strains could reflect differences in their genetic arsenals or biofilm regulatory pathways in response to osmotic stress.

### 3.3. Transcriptomic Analysis of SARB27 and SE016 Strains Under Osmotic Stress

**Figure 2:**
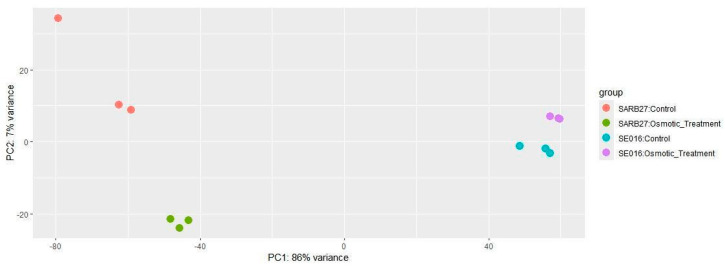
Principal Component Analysis (PCA) of the transcriptomic profiles of SARB27 and SE016 strains. The PCA plot shows the global variance of the RNA-seq data. Each point represents an individual biological replicate, colored by experimental group.

To obtain a global overview of the transcriptomic response, a Principal Component Analysis (PCA) was conducted on the transcriptomic profiles of the SARB27 and SE016 strains. Figure 2 shows the PCA plot, representing the global variance of the RNA-seq data. The clustering of replicates from the same condition and the separation between control and stress conditions, or between strains, the PCA indicates that osmotic stress induces distinct and reproducible changes in gene expression. This supports the reproducibility of the RNA-seq data and indicates distinct transcriptional responses between control and osmotic-stress conditions.

**Figure 3:**
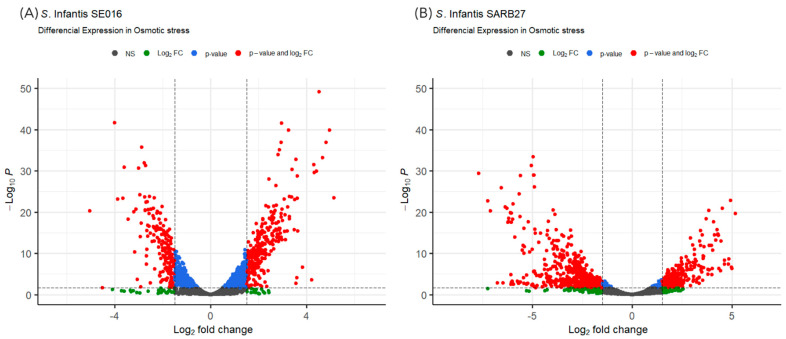
Volcano plot of differential gene expression in response to osmotic stress. The plots show the change in gene expression (Log_2_ Fold Change, *x*-axis) relative to statistical significance (−Log_10_ *p*-value, *y*-axis) for each gene in the transcriptome. The analysis compares osmotic stress with the control for (**A**) strain SE016 and (**B**) strain SARB27. Points represent individual genes, colored according to significance thresholds: red for genes exceeding both the *p*-value threshold (horizontal dashed line) and the expression change threshold (vertical dashed lines); blue for *p*-value only, green for expression change only, and gray for none.

To identify differentially expressed genes (DEGs) in response to osmotic stress, Volcano plots were generated for each strain, comparing the osmotic stress condition with the control condition (Figure 3). The number and magnitude of gene expression changes (Log_2_ Fold Change (LFC)) in these plots are key indicators of the stress response’s intensity. For strain SE016, 426 downregulated and 584 upregulated genes were determined (LFC > |1|, *p*-adj < 0.01), while strain SARB27 exhibited an increase in differentially expressed genes, with 655 downregulated and 559 upregulated genes.

Strain SE016 exhibited fewer differentially expressed genes than SARB27 under osmotic stress, indicating a quantitatively distinct transcriptional response between the two strains. Functional enrichment analyses were subsequently used to explore qualitative differences in the affected pathways.

### 3.4. Gene Ontology (GO) Term Enrichment Analysis for Differentially Expressed Genes

**Figure 4:**
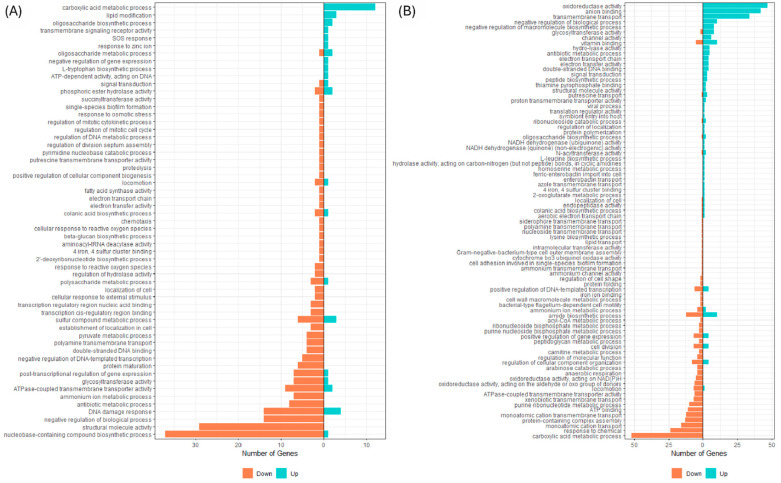
Gene Ontology (GO) term enrichment analysis for differentially expressed genes in response to osmotic stress. The bar charts show the functional categories (GO terms) significantly overrepresented in the sets of differentially expressed genes for (**A**) strain SE016 and (**B**) strain SARB27. The bars indicate the number of genes within each category that were repressed (down, red, left) or induced (up, cyan, right) by osmotic stress. All the terms presented have a *p*-value < 0.01.

To understand the biological functions associated with changes in gene expression, a Gene Ontology (GO) term enrichment analysis was performed for the differentially expressed genes. Functional enrichment analysis revealed that differentially expressed genes were significantly grouped into contrasting Gene Ontology (GO) categories. In strain SE016 (Figure 4A), a significant induction was observed in categories associated with carboxylic acid metabolic processes and polysaccharide and oligosaccharide biosynthetic processes. Conversely, categories related to locomotion, chemotaxis, and the regulation of bacterial-type flagellum-dependent motility were predominantly composed of repressed genes. In comparison, strain SARB27 exhibited more extensive repression across carboxylic acid metabolic processes and lacked significant enrichment in polysaccharide biosynthetic pathways under the same osmotic stress conditions (Figure 4B).

### 3.5. Gene–GO Term Interaction Networks

**Figure 5:**
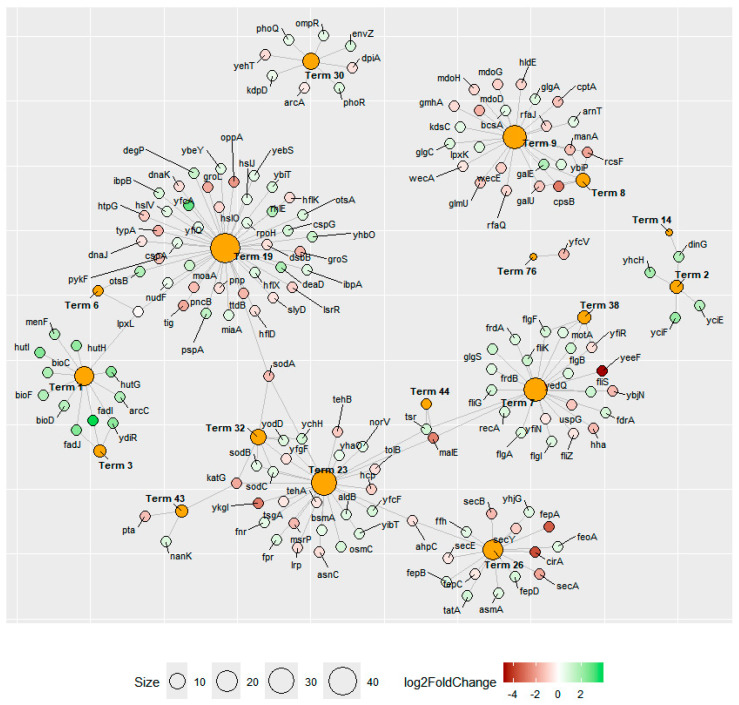
Gene–Concept GO Interaction Network illustrating the relationships between differentially expressed genes and enriched functional categories (GO) in strain SE016 under osmotic stress. In the network, the larger orange nodes represent enriched GO terms, including the following: Term 1—carboxylic acid metabolic process, Term 2—DNA damage response, Term 3—lipid modification, Term 6—oligosaccharide biosynthetic process, Term 8—colanic acid biosynthetic process, Term 9—polysaccharide biosynthetic process, Term 14—polysaccharide metabolic process, Term 19—response to temperature stimulus, Term 23—response to oxygen-containing compound, Term 26—establishment of localization in cell, Term 30—phosphorelay signal transduction system, Term 32—cellular response to reactive oxygen species, Term 38—bacterial-type flagellum-dependent swarming motility, Term 43—antibiotic catabolic process, Term 44—chemotaxis, and Term 76—fimbrial usher porin activity. Smaller nodes represent individual genes, colored according to their expression change (Log_2_FoldChange): red for repression and green for induction. Lines connect genes to the functional categories to which they belong.

The Gene–GO term interaction network for strain SE016 reveals a connection between genes whose expression levels change under osmotic stress (Figure 5). Specifically, the network highlights genes associated with the “Phosphorelay signal transduction system”, such as the two-component system components *ompR* and *envZ*, which are known to be involved in the osmotic stress response. Another notable interaction involves the response to oxygen compounds and reactive oxygen species under oxidative stress caused by high osmotic pressure. Furthermore, a metabolic reorganization of lipids and polysaccharides, as well as carboxylic acid metabolism, can be observed, suggesting a strategic allocation of metabolic resources toward biofilm formation.

According to the Gene–GO term interaction network (Figure 5), genes associated with lipid modifications (Term 3) and DNA damage (Term 2) are all upregulated, while other categories exhibit varied expression patterns. This profile suggests induction of genes associated with cellular damage responses during osmotic stress.

### 3.6. Metabolic Reprogramming and c-di-GMP-Mediated Alternative Biofilm Activation in Response to Osmotic Stress

Comparative transcriptomic analysis revealed that the enhanced biofilm formation and survival of strain SE016 are not driven by constitutive resistance, but by a coordinated transcriptional reprogramming absent in the sensitive strain SARB27 (Table 2).

The osmotic stress response in SE016 was associated with transcriptional changes in genes linked to the OmpR/EnvZ regulatory system, including repression of the ompF porin gene and induction of ompR/envZ. This signaling triggers a critical metabolic brake that prevents cellular toxicity. Whereas strain SARB27 maintained active expression of citrate synthase (*gltA*, LFC = +0.88), suggesting a failed attempt to sustain carbon flux, strain SE016 actively arrested the tricarboxylic acid (TCA) cycle. Specifically, SE016 repressed key genes, including isocitrate dehydrogenase (*icd*, LFC = −1.41) and malate dehydrogenase (*mdh*, LFC = −2.15), consistent with an energy-conservation strategy.

Furthermore, SE016 showed induction of genes associated with anaerobic respiration, including the nitrate reductase operons narGHI and narZWV, suggesting a transcriptional response resembling adaptation to low-oxygen conditions. In contrast, SARB27 failed to activate the *narZ* system, revealing an inability to manage redox balance under high-osmolarity conditions.

Consistent with the genomic approximation for energy-efficient survival, SE016 responded to osmotic shock by maximizing the intracellular accumulation of osmoprotectants through a dual mechanism of transport and conservation. We observed a substantial induction of the gene codifying for the high-affinity *proU* transport system (*proV* and *proW* genes, LFC > 2.7) and the secondary transporter *proP* (LFC = +1.08), that could be associated with the aim of increasing proline and glycine-betaine uptake from the medium. In parallel, the strain activated endogenous trehalose biosynthesis via the induction of the *otsAB* operon (LFC = +1.7).

Crucially, this accumulation was reinforced by the repression of the *putA* gene (LFC = −2.72), encoding proline dehydrogenase. This repression could prevent proline catabolic degradation, ensuring that this amino acid was preserved as a compatible solute for cellular osmotic balance rather than being catabolized as a carbon and nitrogen source.

Notably, the robust biofilm formation observed in strain SE016 under osmotic stress occurred despite significant repression of the gene codifying for the master curli regulator, *csgD* (LFC = −1.41), a well-defined direct effect of OmpR activity under high-osmolarity conditions. To elucidate the alternative mechanism of sessility in SE016, we examined the regulation of c-di-GMP metabolism.

The data revealed that SE016 activates a cellulose-based matrix biosynthesis pathway via specific induction of the diguanylate cyclase *adrA* (LFC = +1.66). Unlike other cyclases, AdrA synthesizes a c-di-GMP pool that allosterically activates the cellulose synthase complex (Bcs); consequently, the structural genes (*bcsA*, *bcsZ*) remained stable and available to drive cellulose polymerization. This strategy allows SE016 to rapidly generate a protective matrix without the energetic cost of synthesizing proteinaceous fimbriae. Indeed, the fimbrial clusters (*kfl* and *lpf*) of the pESI megaplasmid present in SE016, as well as the curli machinery, are repressed by high-osmotic conditions. This confirms that the resistance phenotype under osmotic stress is driven by a chromosomal response (cellulose) rather than by plasmid-encoded adhesins.

Additionally, the intracellular levels of c-di-GMP were determined (pg/μL) at 8, 16 and 24 h, in the strains SARB27 and SE016 in the conditions: MgM (control) and MgM supplemented with 15% sucrose (osmotic stress). There are statistical differences in the c-di-GMP concentration, correlated to the resistance phenotype (SE016) (Supplementary Figures S2 and S3). These results suggest that *Salmonella* responds to osmotic stress through a coordinated adaptive transcriptional response involving metabolic arrest, osmoprotectant conservation, anaerobic respiration, and AdrA/c-di-GMP–mediated cellulose biofilm formation, ultimately promoting an energy-efficient and stress-resilient phenotype.

## 4. Discussion

The global increase in the prevalence of *S.* Infantis in human salmonellosis cases is well documented, establishing it as one of the non-typhoidal serovars of greatest concern [38]. This epidemiological trend is intrinsically linked to the food production chain; specifically, the poultry industry has been identified as the primary reservoir and major source of contamination for humans [39]. Chicken meat and its derivatives are predominant vehicles for the transmission of this pathogen, underscoring the importance of understanding its survival mechanisms in this specific ecological niche [40].

The recurrent persistence of *Salmonella* in these facilities, despite control measures, is a widely recognized phenomenon, largely attributed to Salmonella’s ability to respond to these types of stress by forming biofilms [40]. The biofilm matrix acts as a physical shield protecting bacteria from desiccation, radiation, antimicrobial agents, and the host immune system, making cells within the biofilm more resistant to disinfectants than their planktonic counterparts. This survival strategy is fundamental for contaminating key surfaces such as water systems, feed lines, and processing equipment.

This study highlights the relationship between environmental stress and bacterial persistence. The phenotypic results (Figure 1) demonstrate that the poultry-derived isolate SE016, when subjected to simulated osmotic stress (15% sucrose), exhibits a marked reduction in the expression of genes related to flagellar motility and a concomitant significant increase in biofilm formation. This behavior contrasts with that of the control strain SARB27, suggesting that this response is a distinctive characteristic of an isolate adapted to the constant stress of the industrial environment. Furthermore, the phenotypic transition from a motile to a sessile lifestyle, observed in response to osmotic stress, is underpinned by a massive and precise reorganization of gene expression associated with adaptation strategies to withstand disinfection protocols. The transcriptomic analysis performed in this study provides direct molecular evidence of this cellular paradigm shift, revealing a coordinated genetic program that shapes the behavior of *S.* Infantis SE016 to adjust the transcriptomic response.

Principal Component Analysis (PCA) of gene expression profiles (Figure 2) demonstrates that osmotic stress induces a global, distinctive, and reproducible change in the transcriptome of strain SE016. The clear separation between the control and osmotic stress groups in the PCA space indicates that the cell activates a transcriptomic response program, validating the biological relevance of the applied stimulus. The magnitude of this response is reflected in a significant number of differentially expressed genes (Figure 3). While SE016 exhibits a transcriptomic adaptation pattern, the magnitude of this transcriptomic response is consistent with the presence of a specialized genetic circuit in strain SE016, the observed transcriptional profile may reflect a cross-protection response associated with oxidative stress adaptation [41].

Gene Ontology (GO) term enrichment analysis (Figure 4 and Figure 5) contributes to the understanding of the transcriptomic response. The results confirm a coordinated transition from motility to sessility. Specifically, genes regulated in strain SE016 under osmotic stress are significantly enriched in functional categories such as “carboxylic acid metabolic process,” “polysaccharide biosynthetic process,” “lipid modification,” and “chemotaxis”. This concerted repression of genes associated with the motility apparatus correlates with the phenotypic reduction in motility observed in previous assays (Figure 1), suggesting that the reprogramming aims to avoid singular modifications to the cell membrane. This shift in motility is a conserved strategy in biofilm formation, as it allows the cell to conserve energy and metabolic resources to invest in building the biofilm matrix [42]. Additionally, enrichment of terms associated with thermal stimulus could be linked to a response to other stressors such as osmotic pressure, in which gene redundancy and overlap between different stress regulons may play a significant role in enhancing cellular resilience; however, this is yet to be determined and further studies should address it.

The enrichment of functional categories such as “Response to oxygen-containing compounds” and “Cellular response to reactive oxygen species” in SE016 points toward an integrated transcriptomic response. This profile reflects a potential anticipatory strategy where master regulators, including the OmpR/EnvZ system [23] and RpoS [43], coordinate a response to secondary oxidative damage induced by hyperosmotic conditions. While direct cross-protection against oxidative disinfectants was not explicitly measured, these findings align with established models of stress-induced cross-tolerance, documented as a driver of persistence of *Salmonella* in industrial environments. Physiologically, hyperosmolarity-induced cellular dehydration can disrupt the electron transport chain, resulting in electron leakage and the endogenous generation of superoxide and hydrogen peroxide [44]. In this context, whereas the sensitive strain SARB27 exhibited the induction of antioxidant genes, the downregulation of TCA cycle-associated genes suggests a possible reduction in tricarboxylic acid cycle activity under osmotic stress. This suggests that SE016 may activate pathways associated with oxidative-stress mitigation under osmotic stress. This observation has significant implications for bacterial persistence within the poultry industry. Many disinfectants used in processing function by generating oxidative stress [45]. If the osmotic stress generated during surface desiccation or within high-concentration residues activates molecular systems to avoid or mitigate oxidative stress, the bacterium may become phenotypically resistant to oxidative disinfectants. This stress-induced cross-tolerance could explain why strains SE016 and *S.* Infantis persisted even after standard industrial disinfection protocols.

Our findings demonstrate that the response of S. Infantis to disinfectants depends on both the type of agent and the physiological state. The greater growth capacity observed in biofilms for strain SE016 when exposed to oxidizing disinfectants, such as peracetic acid and sodium hypochlorite, suggests a more effective response to damage mediated by reactive oxygen species, which affect lipids, proteins, and DNA [46,47]. This difference is not observed under planktonic conditions, where MIC values are comparable or even lower for SE016, indicating that the differential behavior is associated with the biofilm context.

Biofilm organization generates microenvironments with gradients of oxygen, pH, and compound diffusion that modulate disinfectant activity and promote bacterial persistence [18,19,33]. In this context, the integration of oxidative, acid, and osmotic stress responses is critical. Global regulators such as OmpR coordinate these responses, enabling adaptation to changes in pH and osmolarity and contributing to the response to disinfectants [15,23,48].

In contrast, non-oxidizing disinfectants such as quaternary ammonium compounds and glutaraldehyde did not show marked differences between strains, suggesting that their mechanisms of action, which primarily target membranes and proteins, respectively, do not elicit differential responses in biofilms under the conditions tested [49,50].

Overall, these findings highlight that the interplay between multiple stress responses and biofilm organization is a key determinant of S. Infantis gene expression patterns in response to disinfectants, particularly in industrial environments.

Evaluating the differences between the sensitive strain SARB27 and SE016 revealed a fundamental divergence in central metabolic management. Whereas SARB27 attempted to maintain normal metabolic activity, SE016 reduced the expression of genes associated with the tricarboxylic acid (TCA) cycle. The repression of key genes such as isocitrate dehydrogenase (*icd*) and malate dehydrogenase (*mdh*), coupled with the downregulation of citrate synthase (*gltA*), constitutes a metabolic brake [23]. Under acute osmotic stress conditions, sustaining the TCA cycle at normal rates would be metabolically detrimental. The TCA cycle is the primary source of NADH, which, when processed via the respiratory chain, is a major source of reactive oxygen species (ROS). Downregulation of genes associated with the TCA cycle may reduce ROS-associated stress, although ROS levels and oxidative damage were not directly measured in this study. This is reflected in the observed non-activation and repression of antioxidant genes in SE016 [51]. Furthermore, this metabolic pause prevents the depletion of intermediate metabolites required for emergency biosynthetic pathways. For instance, acetyl-CoA can be diverted toward fatty acid synthesis for membrane remodeling rather than oxidation, a shift reflected in the enrichment of genes associated with “Lipid modification” and “Carboxylic acid metabolic process” in SE016. The transcriptomic profile of strain SE016 shows a significant down-regulation of *gltA,* concurrent with activation of the OmpR/EnvZ system, consistent with validated regulatory models in *Salmonella*. It has been demonstrated that OmpR functions as a direct transcriptional repressor of *gltA*, which could help modulate carbon flux to prioritize stress adaptation over basal aerobic metabolism during osmotic challenges [23]. Thus, the observed differential expression in SE016 likely reflects the execution of this established signaling circuit to optimize cellular resources under hyperosmotic conditions.

We observed the induction of the nitrate reductase operons *narGHI* and *narZWV* in SE016 under aerobic experimental conditions. Based on the observed transcriptomic profile, it is plausible to infer that the bacterium perceives acute osmotic stress as a signal analogous to hypoxia, as evidenced by the induction of genes related to anaerobic respiration and alternative metabolic pathways. This transcriptional reprogramming likely provides metabolic flexibility under high osmolarity, supporting sustained viability in diverse environmental niches. This inference aligns with characterized mechanisms in which OmpR integrates osmotic signals to modulate alternative metabolic pathways, such as activating *narZ* to manage cellular stress [23,48].

Nitrate reductase A (NarGHI) enables *Salmonella* to utilize nitrate as a terminal electron acceptor, generating the proton motive force essential for ATP synthesis [52]. In the context of infection, this capability is crucial for systemic host invasion. In the poultry industry, the ability to switch from aerobic cellular respiration to nitrate respiration confers a significant survival advantage. Oxygen is rapidly depleted within the interior of a developing biofilm, creating anoxic microenvironments. Consequently, the activation of *narGHI* and *narZWV* during osmotic stress, these transcriptional changes may contribute to adaptation to conditions resembling those encountered in deeper biofilm layers.

Furthermore, the regulation of osmoprotectants in SE016 demonstrates a superior adaptive response relative to the sensitive strain SARB27. SE016 exhibited strong induction of the osmotically inducible high-affinity transport system *proU* and the low-affinity transporter *proP*, thereby maximizing the uptake of proline and glycine-betaine from the environment [53]. Accumulation of these compounds to high intracellular concentrations counterbalances external osmotic pressure without perturbing cellular machinery [54]. Additionally, this accumulation was reinforced by the repression of *putA*, which encodes a proline dehydrogenase that converts proline to glutamate for use as a carbon and nitrogen source. Under osmotic stress, *putA* repression is crucial for maintaining cellular turgor [55].

Moreover, the induction of *otsAB* for trehalose synthesis provides an additional layer of protection. Trehalose functions not only as an osmolyte but also as a chemical chaperone, stabilizing protein structures and cell membranes during stress conditions [56]. The metabolic prioritization toward trehalose and proline accumulation observed in SE016 reflects fundamental mechanisms of long-term evasion of hyperosmotic stress [20]. In environments with severe water restriction, Salmonella utilizes these solutes to stabilize protein structures and lipid membranes against peroxidation induced by reactive oxygen species [22]. This redundancy in protective systems in response to osmotic stress renders *S.* Infantis strain SE016 highly resilient to fluctuations in osmotic pressure.

Regarding biofilm formation, it is well established in *Salmonella* that the master regulator CsgD activates the transcription of the curli fimbriae operon (*csgBAC*) and the diguanylate cyclase gene *adrA*, which produces the c-di-GMP required for cellulose synthase activation [24]. However, under high-osmolarity conditions, the phosphorylated global regulator OmpR (OmpR-P) binds to low-affinity sites within the *csgD* promoter, effectively repressing its expression [24]. Theoretically, this repression should abolish biofilm formation.

Nevertheless, strain SE016 formed a robust biofilm despite the observed downregulation of *csgD*, a finding that confirms the canonical OmpR-mediated repression mechanism remains intact under high osmolarity while highlighting a divergence in the phenotypic outcome. This phenotype was associated with *adrA* induction occurring independently of detectable *csgD* upregulation.

AdrA is a diguanylate cyclase responsible for c-di-GMP synthesis. Unlike other cyclases that contribute to a global cytosolic pool of c-di-GMP, AdrA interacts specifically with the cellulose synthase complex (Bcs), providing efficient, localized activation of cellulose production [57]. The induction of *adrA* in the absence of significant CsgD levels suggests the involvement of an alternative regulatory pathway. It has been reported that other factors, such as Crl, which potentiates RpoS activity, can directly stimulate *adrA* transcription under specific stress conditions, thereby partially decoupling it from the CsgD activation pathway.

The transcriptional and phenotypic data are consistent with increased cellulose production contributing to the biofilm matrix composition [18]. This cellulose-rich matrix proves highly advantageous under osmotic stress. Cellulose is a highly hydrophilic polymer that can retain water, thereby protecting cells from desiccation and concentrating nutrients and osmoprotectants within the immediate cellular microenvironment [58]. Furthermore, cellulose confers superior resistance to specific disinfectants and mechanical stress relative to proteinaceous fimbriae [59].

## 5. Conclusions

This study provides insight into transcriptional and phenotypic responses to osmotic stress in the poultry-derived *Salmonella enterica* serovar Infantis strain SE016. The strain showed transcriptional and phenotypic responses consistent with activation of stress-associated regulatory pathways, including pathways linked to OmpR/EnvZ. These changes are consistent with reduced central metabolic activity and possible energy conservation under osmotic stress. These changes facilitate anaerobic metabolism as an emergency energy contingency (nitrate respiration) and trigger a cellulose-based biofilm construction pathway via the CsgD-independent activation of AdrA. This phenotypic plasticity highlights the significant adaptive potential of *S.* Infantis within the global food supply chain, suggesting that its capacity for persistence may extend beyond the specific context of poultry production.

## 6. Limitations and Future Directions

This study has several limitations. First, the detailed transcriptomic and phenotypic analyses focused primarily on one poultry-derived *S.* Infantis strain, SE016, and therefore the findings should not be generalized to all *S.* Infantis lineages without broader strain-level validation. Second, 15% sucrose was used as a controlled experimental model of osmotic stress and does not fully reproduce the complexity of poultry-processing environments, where bacteria may be exposed to combined chemical, oxidative, desiccation, nutrient, temperature, and surface-associated stresses. Third, although RNA-seq identified transcriptional changes in pathways associated with OmpR/EnvZ, c-di-GMP signaling, osmoprotection, nitrate respiration, and biofilm formation, these results do not by themselves demonstrate causality. Future studies should include functional validation using targeted mutants or complementation assays, direct quantification of ROS, cellulose and/or extracellular matrix components, proteomic or metabolomic analyses, and testing on industrially relevant surfaces under combined stress and disinfectant-exposure conditions. Such studies would help determine whether the transcriptional patterns observed here translate into stable phenotypic advantages in poultry-processing environments.

## Figures and Tables

**Table 1:** Disinfectants, stock and application concentrations, and exposure time.

Disinfectant	Stock Concentration	Application Concentration	Exposure Time
Quaternary ammonium	10%	450 ppm	10 min
Peracetic acid	15%	300 ppm	10 min
Glutaraldehyde	2%	200 ppm	10 min
Sodium hypochlorite	10%	200 ppm	10 min

**Table 2:** Comparative transcriptomic analysis of key genes in response to osmotic stress.

Functional Category	Gene	Function	SE016 Log2FC	SE016 *p*adj	SARB27 Log2FC	SARB27 *p*adj
**Regulation (OmpR)**	*ompR*	Response regulator	0.63	0.014	0.67	0.23 (ns)
	*envZ*	Kinase sensor	0.88	0.012	−0.34	0.60 (ns)
	*ompF*	Major porine (entrance)	−3.56	<0.001	−2.72	<0.001
**Central Metabolism**	*gltA*	Citrate synthase (TCA)	−0.30	0.022	0.89	0.16 (ns)
	*icd*	Isocitrate DH (TCA)	−1.41	<0.001	1.20	0.10 (ns)
	*mdh*	Malate DH (TCA)	−2.15	<0.001	−2.05	0.001
**Anaerobic Respiration**	*narG*	Nitrate reductase A	1.46	<0.001	1.71	0.047
	*narZ*	Nitrate reductase Z (Aux)	1.07	<0.001	0.02	0.98 (ns)
**Osmoprotection**	*proV*	Transp. ProU (ATPasa)	2.88	<0.001	0.15	0.86 (ns)
	*otsB*	Trehalose Synthesis	1.70	<0.001	1.44	0.002
	*putA*	Trehalose Synthesis	−2.72	<0.001	−2.58	<0.001
**Biofilm**	*csgD*	Curli Master Regulator	−1.41	<0.001	−2.04	0.007
	*adrA*	DGC (c-di-GMP Synthesis)	0.66	0.039	0.94	0.11 (ns)
	*bcsA*	Cellulose synthase	0.55	0.047	−0.71	0.16 (ns)
**pESI fimbriae**	*klf operon*	K88-like Fimbriae	−0.97	<0.01	N/D	-
	*lpf operon*	Long Polar Fimbriae	−0.60	0.10 (ns)	N/D	-

note: “ns” is not significant; “N/D” no data reported in the studied genome.

## Data Availability

The original contributions presented in this study are included in the article/Supplementary Material. Further inquiries can be directed to the corresponding author.

## Supplementary Materials

The following supporting information can be downloaded at: https://www.mdpi.com/article/10.3390/foods15111938/s1, Figure S1: Survival challenge of Salmonella Infantis strains SARB27 and SE016 against common disinfectants; Figure S2: Quantification of intracellular c-di-GMP in the planktonic and sessile fractions of Salmonella Infantis under control and osmotic stress conditions; Figure S3: Quantification of intracellular c-di-GMP levels in the planktonic phase of S. infantis strains; Table S1: Minimum inhibitory concentration of *Salmonella* Infantis SARB26 and SE016 against industrial disinfectants.
