# Supplementary material for: Osmotic Stress Adaptation of Poultry-Associated Salmonella Infantis and Its Implications for Food Safety
Source: Foods. 2026 May 31;15(11):1938. doi: 10.3390/foods15111938 (PMC13257351; doi:10.3390/foods15111938)
Supplement: Supplementary file 1 [file foods-15-01938-s001.zip › Supplementary Figure S3.pdf]

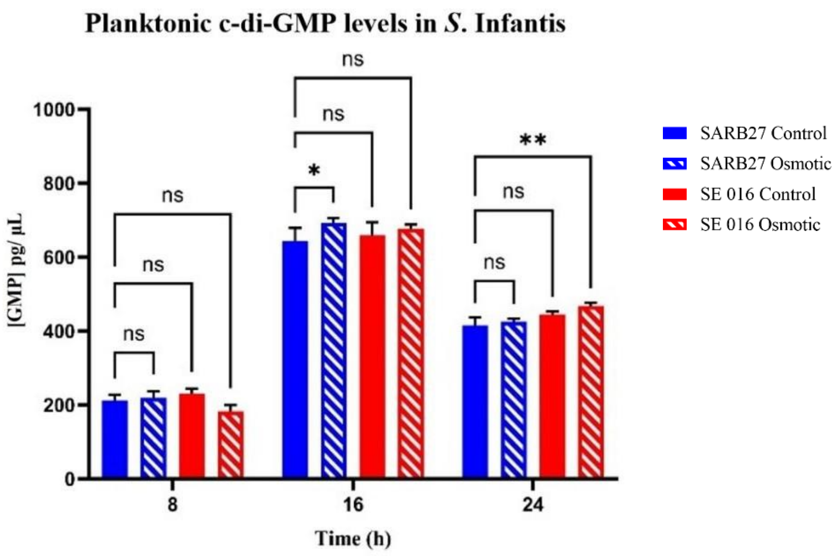

Supplementary Figure 3: Quantification of intracellular c-di-GMP levels in the planktonic phase of *S.* infantis strains.

c-di-GMP levels (pg/µL) were quantified at 8, 16, and 24 hours in strains SARB27 and SE016 under MgM (control) and MgM supplemented with 15% sucrose (osmotic) conditions. The assay was performed in parallel with biofilm growth kinetics. Significant differences in c-di-GMP concentrations were observed, correlating with the resistance phenotype (SE016). Significance was determined using two-way ANOVA (ns: not significant, * $p < 0.05$; ** $p < 0.01$) (n=6).
