# Supplementary material for: Osmotic Stress Adaptation of Poultry-Associated Salmonella Infantis and Its Implications for Food Safety
Source: Foods. 2026 May 31;15(11):1938. doi: 10.3390/foods15111938 (PMC13257351; doi:10.3390/foods15111938)
Supplement: Supplementary file 1 [file foods-15-01938-s001.zip › Supplementary Figure S1.pdf]

Supplementary Figure S1.

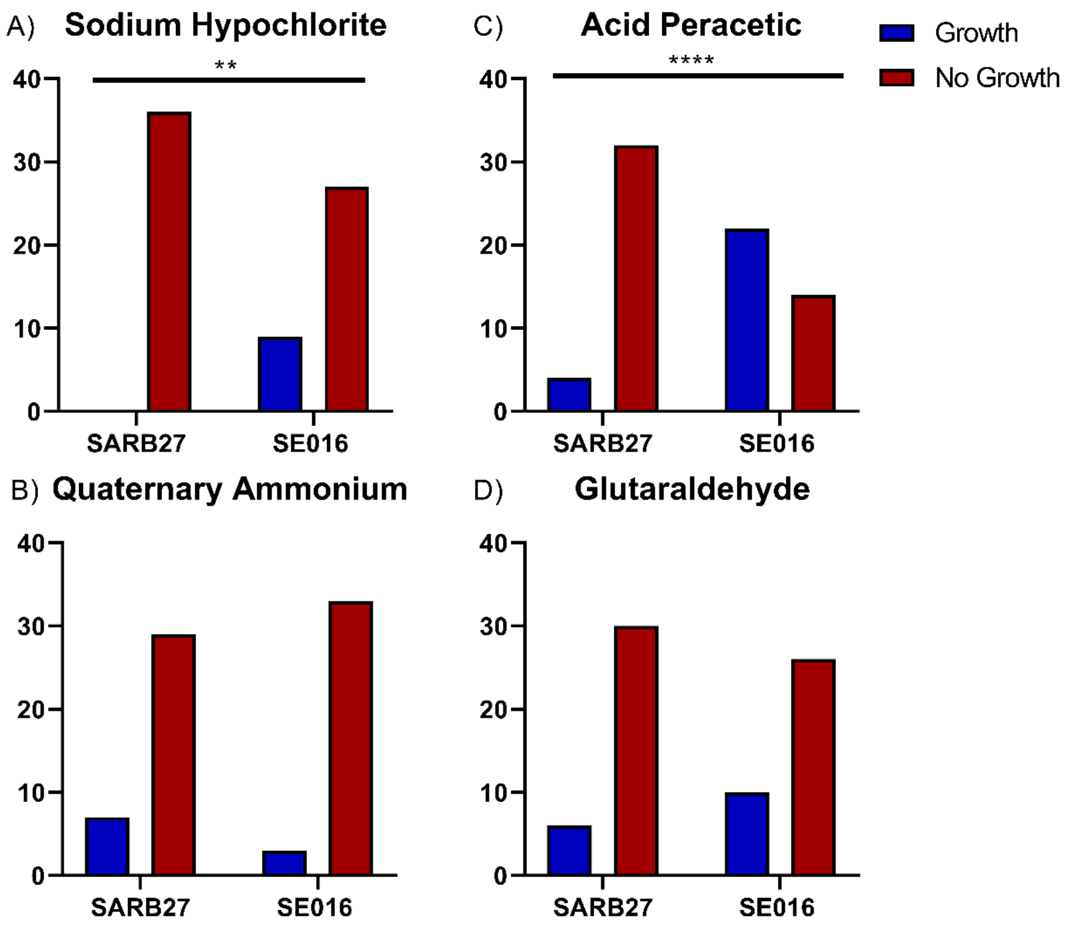

Supplemtary figure S1. Survival challenge of *Salmonella* Infantis strains SARB27 and SE016 against common disinfectants. The graphs display the frequency of growth (blue) and no growth (red) for each strain after exposure to (A) Sodium Hypochlorite, (B) Quaternary Ammonium, (C) Peracetic Acid, and (D) Glutaraldehyde. Results are based on n=36 replicates per strain. Statistical significance was determined using Fisher's exact test. Asterisks indicate significant differences in survival rates between strains ($p < 0.01$; ****$p < 0.0001$).
