# Supplementary material for: Osmotic Stress Adaptation of Poultry-Associated Salmonella Infantis and Its Implications for Food Safety
Source: Foods. 2026 May 31;15(11):1938. doi: 10.3390/foods15111938 (PMC13257351; doi:10.3390/foods15111938)
Supplement: Supplementary file 1 [file foods-15-01938-s001.zip › Supplementary Figure S2.pdf]

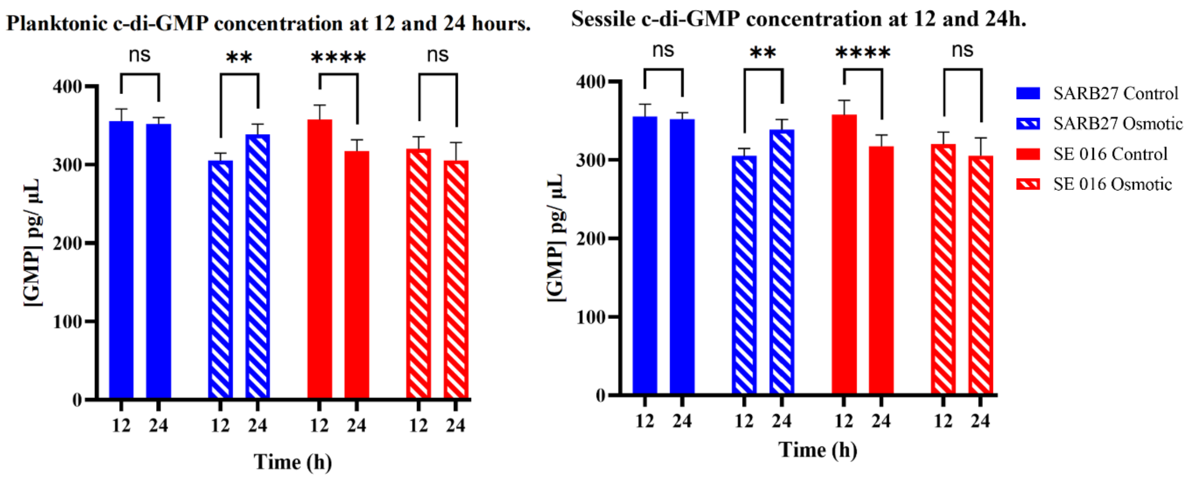

Supplementary Figure S2: Quantification of intracellular c-di-GMP in the planktonic and sessile fractions of *Salmonella* Infantis under control and osmotic stress conditions.

The concentration of c-di-GMP (pg/µL) was determined at 12 and 24h in the reference strain SARB27 and the resistant strain SE016, cultured in MgM medium (control) and MgM + 15% sucrose (osmotic). To this end, the planktonic (left panel) and sessile (right panel) fractions were separated, and intracellular c-di-GMP was quantified using the Cyclic-di-GMP Assay Kit (Lucerna). In the sessile fraction, SARB27 under osmotic conditions showed a significant increase in c-di-GMP between 12 and 24 h, while SE016 under control conditions exhibited a significant decrease over the same interval. In the planktonic fraction, only SARB27 under control conditions showed a significant decrease in c-di-GMP at 24 h. Data are presented as mean ± SD, and significance was determined using two-way ANOVA (ns: not significant; ** $p < 0.01$; **** $p < 0.0001$) ($n = 6$).
